# Crystal engineering and **IUCrJ**

**DOI:** 10.1107/S2052252515024100

**Published:** 2016-01-01

**Authors:** Gautam R. Desiraju

**Affiliations:** a Solid State and Structural Chemistry Unit, Indian Institute of Science, Bangalore 560 012, India

**Keywords:** Editorial, crystal engineering

## Abstract

Crystal engineering is discussed, along with its three key concepts: crystal packing; the design of solids; and physical and chemical properties.

Crystal engineering has grown over time, with its practitioners now seeking specific answers to specialized questions. How does a molecular crystal nucleate and then grow? Can its structure be predicted computationally? Can one design a crystal structure with knowledge-based inputs? Can a crystal structure be considered as a collection of modular entities which represent its microcosms? What properties are characteristic of the crystal as a whole rather than of its constituent molecules? Can these properties be designed and is property design different from structure design? Can one predict if a given compound will have polymorphs and pseudopolymorphs? Can one design the structures of multi-component crystals in which each component is a solid when taken separately under ambient conditions? All these issues connect through the structural landscape of crystals and the exploration of this landscape, that is crystallization. The subject of crystal engineering covers not only purely organic solids but also organometallics and more significantly the metal organic framework solids (MOFs) or coordination polymers as they are also called.

Crystal engineering evolved from an intersection of crystallography and chemistry, especially after the 1970s when it became much easier to determine crystal structures of small molecule solids. Today, crystal engineering employs crystallography, spectroscopy and computation; around 50 papers have appeared in **IUCrJ** that highlight all three approaches (see, for example, Aakeröy *et al.*, 2015 ▸; Nalla *et al.*, 2015 ▸; Gándara & Bennett, 2014 ▸; Bolla *et al.*, 2015 ▸; Lecomte *et al.*, 2015 ▸). It is interesting that the subject is seen as mature enough for ICSU to organize a project with IUCr and IUPAC to delineate a set of nomenclature guidelines for crystal engineering. Is it important to have a definition of the term *crystal engineering* itself? This question resurfaces with regularity. I provided a working definition back in 1989 (Desiraju, 1989 ▸) and termed it as the ‘understanding of intermolecular interactions in the context of crystal packing and the utilization of such understanding in the design of new solids with desired physical and chemical properties’. This wording seems to have stood the test of time as it identifies the three concepts that are of note in the subject: crystal packing; the design of solids; physical and chemical properties.

Crystal packing is closely connected with the nature of intermolecular interactions, their strength, their directionalities and their distance dependence properties. These three attributes determine how and to what degree of importance an interaction manifests itself in the packing of molecules in a crystal. It is fair to say that ‘the understanding of intermolecular interactions in the context of crystal packing’ has largely to do with understanding these three particular features. Papers have appeared in the journal on hydrogen bonding, halogen bonding and van der Waals interactions. The related area of charge density studies is closely connected to the study of intermolecular interactions and the importance of charge density studies to crystal engineering is now acknowledged. One may also ask if it is an over simplification to consider a crystal as an ensemble of two-body interactions. In the limit, the molecular crystal is a holistic entity. Each crystal structure is, rigorously speaking, a different story. Why then does the modular approach with supramolecular synthons work so well in crystal design?

The design of crystal structures calls for some element of predictability in the manner in which one molecule recognizes another during assembly. At the core of the supramolecular synthon approach to crystal design is the argument that one functional group in a molecule recognizes another functional group in the same or different molecule in a particular way. This recognition pattern defines the synthon. The role of the supramolecular synthon in crystal engineering may be likened to that of the molecular synthon in organic synthesis because both types of synthon, molecular and supramolecular, arise from kinetically preferred events. Logic driven retrosynthesis may be used to design organic crystal structures. A similar retrosynthetic analysis may be applied to the design of coordination polymers and MOF compounds. When the functional groups that recognize each other arise from the same type of molecule, the result is a single component crystal. When they are from different chemical entities, the result is a multi-component crystal or a cocrystal. One hears of the term ‘cocrystal engineering’ here and there but it is important to note that the chemistry that underlies molecular recognition and pattern conservation through supramolecular synthons is exactly the same, whether or not a single component or multi-component crystal is sought to be designed.

Property design is the third and final stage in crystal engineering. It is interesting to note, around three decades after the term *crystal engineering* itself entered the chemical and crystallographic literature in a general way, that each of the three stages in the development of the subject, crystal packing analysis, crystal design strategies and targeting of properties needed a certain degree of maturity of the earlier stages before they could develop systematically. It was certainly unfair to expect any serious property engineering say 15 or 20 years ago, although there was progress in the design of non-centrosymmetric crystals for optical devices. Today there is an explosion of activity in the design of crystal properties, ranging from gas absorption and catalysis applications in MOFs to mechanical, photochemical and photophysical properties for pure organics. All of us know that structure and properties are connected. One can think of form and function in the macromolecular crystallography context. But what exactly is the connection? Sometimes, there is a clear connection so that a particular change in the structure within say, a given family of crystals leads to a predictable change in the property. However, there are other cases in which a very small change in the structure may lead to a large change in the property. This is certainly true of electronic and mechanical properties. This also leads to the idea as to whether or not property design must go through structure design. Structural modulations are often continuous. Desirable properties sometimes need to be of the ‘on–off’ variety. How does one relate these seemingly contradictory requirements?

It is in the above context that prospective authors need to look at **IUCrJ** as a location to place their latest work. It is a measure of the changing scope and meaning of the terms *crystal*, *crystallography* and *crystallographer* that the IUCr has selected the theme of *Chemistry and Crystal Engineering* as one of the areas to be covered by its flagship journal. The journal actively seeks papers in crystal engineering that are directed to the chemist-crystallographer or to any chemist with a strong interest in the overall domain of structure. One has gone beyond the stage where one would discuss at length what chemistry was and what crystallography was and how these worlds intersected or why they did not. Answers to complex questions require a flexible approach and **IUCrJ** is a journal which has now begun the tradition of publishing papers that seek to make and change opinion in this open and broad minded subject.
